# HBsAg as an important predictor of HBeAg seroconversion following antiviral treatment for HBeAg-positive chronic hepatitis B patients

**DOI:** 10.1186/1479-5876-12-183

**Published:** 2014-06-25

**Authors:** Jiezuan Yang, Jiajia Chen, Ping Ye, Linfeng Jin, Wei Wu, Guoping Sheng, Lan-Juan Li

**Affiliations:** 1 State Key Laboratory for Diagnosis and Treatment of Infectious Diseases, the First Affiliated Hospital, College of Medicine, Zhejiang University, Hangzhou, China; 2 Collaborative Innovation Center for Diagnosis and Treatment of Infectious Diseases, Zhejiang University, Hangzhou, China

**Keywords:** Chronic hepatitis B, Antiviral treatment, HBsAg quantitation, HBeAg seroconversion, Prognostic indicator

## Abstract

**Background:**

Serum quantitative hepatitis B surface antigen (HBsAg) levels may be an important predictor of hepatitis B e antigen (HBeAg) seroconversion (SC) in HBeAg-positive chronic hepatitis B (CHB) patients during antiviral treatment. The pattern of HBsAg variation in CHB patients either with or without SC following tenofovir disoproxil fumarate (TDF) treatment is not clearly understood.

**Methods:**

Twenty patients with full experimental data were enrolled, and liver biochemistry, serum HBV DNA, and circulating CD4^+^CD25^+^ regulatory T cell (Treg) levels were determined at baseline and every 12weeks after the initiation of TDF treatment (for a total of 96weeks). In addition, the relationship between HBsAg or HBeAg and alanine aminotransferase (ALT), HBV DNA and Treg levels in SC and non-SC patients was analyzed.

**Results:**

In all, 9 patients had undergone HBeAg seroconversion by week 72 of TDF treatment, and biochemical and virological indexes and Treg percentages declined to normal levels. Furthermore, the positive correlation between HBsAg and ALT, HBV DNA and Treg levels was significant for SC patients, but not for non-SC patients. However, for HBeAg, significant positive correlations were or not observed for both SC and non-SC patients.

**Conclusions:**

The quantitation of HBsAg is a more useful indicator than HBeAg for distinguishing SC and non-SC patients during TDF treatment. Moreover, HBsAg may be related to immune regulatory property of CHB patients during antiviral treatment.

## Background

Hepatitis B virus (HBV) infection remains a serious health problem that currently affects approximately 350 to 400 million people worldwide. Chronic hepatitis B (CHB) is closely association with the morbidity of liver cirrhosis, liver failure, and hepatocellular carcinoma (HCC) [1]. Antiviral therapy against HBV plays a pivotal role in determining the outcome of CHB. Effective antiviral therapy for CHB would achieve control of viral replication, ALT normalization, HBeAg loss and seroconversion, and a small number of patients may even achieve HBsAg clearance and seroconversion [2,3]. HBeAg seroconversion (SC) is associated with a reduced incidence of progressive liver inflammation, cirrhosis, and failure and HCC [4-6]. CHB patients undergoing SC usually become inactive HBsAg carriers [5]. However, during antiviral therapy, CHB patients who have undergone the potent suppression of HBV replication do not always induce SC, even though liver inflammation is controlled. Patients who do not experience SC frequently develop reactivation with high levels of HBV replication, and liver inflammation resumes when antiviral drugs are stopped [7].

The covalently closed circular DNA (cccDNA) of HBV plays a major role in viral persistence, and its clearance is thought to be the limiting factor for the elimination of infection. Serum HBsAg levels are known to reflect the presence of cccDNA in the liver, and a reduction in HBsAg levels correlates well with cccDNA levels [8]. HBsAg clearance from the serum approximates clinical cure and is associated with improved survival [2]. The dynamics of HBsAg decline have recently been described in patients treated with standard interferon (IFN), pegylated interferon (PEG-IFN), entecavir (ENV), and adefovir (ADV) monotherapy [9-11]. However, the dynamic pattern of serum HBsAg levels in HBeAg-positive CHB patients who have or have not undergone SC following TDF treatment has not been well described. In addition, the relationship between variations in HBsAg levels and ALT, HBV DNA, and circulation Treg percentages have poorly addressed as well [12].

In the present study, we aimed to determine the dynamic profile of serum HBsAg levels in HBeAg-positive CHB patients who did or did not undergo HBeAg seroconversion following TDF therapy, and its correlation with biochemical, virological and immunological parameters during the treatment period.

## Materials and methods

### Subjects and blood samples

A total of 78 outpatients with CHB were enrolled from the Department of Infectious Disease, the First Affiliated Hospital, College of Medicine, Zhejiang University between November 2011 and February 2012. All patients were seropositive for HBsAg and HBeAg. They were 57 men and 21 women (age, mean ± STD, 33.1 ± 8.2 years) without concurrent other infectious diseases and autoimmune liver diseases. At baseline, their HBV-DNA loads were more than 1 × 10^6^ copies/ml (range, 1.5 × 10^7^ – 6.2 × 10^9^ copies/ml) and ALT levels were twice (range, 112 – 452 U/L) the upper limit of the normal levels. The average level of HBsAg is 3.75 ± 0.68 (log10, IU/ml, mean ± STD). For the following reasons, the enrolled CHB patients could not be contacted (31 patients), switched to other nucleotide analogues (entecavir, adefovir) treatment (15 patients) due to the cost, or dropped out during the TDF treatment (12 patients) for unknown reasons, ultimately, only 20 CHB patients were followed serially with protocol visits for 96 weeks continuously during the course of TDF treatment with a daily dose of 300 mg.

The twenty HBeAg-positive CHB patients were classified into SC (n = 9) or non-SC (n = 11) groups, depending on whether they had undergone SC by week 72 of TDF treatment. Subjects with CHB fulfilled the definitions from Hou’s report [13] and The guideline of prevention and treatment for chronic hepatitis B (2010 version) [14]. In addition, exclusion criteria were described in our previous report [15]. Our study conformed to the ethical guidelines of the Declaration of Helsinki, and informed consent was obtained from all patients prior to enrollment. The First Affiliated Hospital, College of Medicine, Zhejiang University medical ethics committee approved all procedures involving human subjects.

### Assessment of biochemical, serological and virological parameters

Patients visited the outpatient clinic every 12weeks for routine examination and laboratory assessment. Routine liver biochemistry, serological HBV parameters, and quantitation of HBeAg and HBsAg levels were performed at baseline and during all follow-up visits. Serum ALT, Albumin (ALB), and total Bilirubin (TBiL) were determined using an automatic biochemical analyzer (AEROSET, Abbott, Chicago, IL, USA). Determination of HBeAg and HBeAb (anti-HBe) status was performed using a commercially available enzyme immunoassay. For measurement of HBsAg and HBsAb, samples were analyzed using commercial enzyme immunoassay kits (Kewei Diagnostic, Beijing, China) according to the manufacturer’s instructions. The serum HBV DNA level was quantified using a Roche second-generation real-time PCR machine (Cobas Ampfiprep/Cobes Taclman) with a detection limit of 116 copies/ml according to the manufacturer’s instruction. HBV genotypes were determined with sequence detection via PCR, and PCR products were directly sequenced with an HBV Genotype Real Time PCR kit (ZJ Bio-Tech, Shanghai, China) and run on a MegaBACE™ 500 according to the manufacturer’s instructions, as has been previously described in more detail [16].

The above indicators were quantified in the clinical laboratory center of the First Affiliated Hospital, College of Medicine, Zhejiang University.

### Flow cytometric analysis of Treg percentage

The percentage of CD4^+^CD25^+^ regulatory T cells (Treg) in CD4^+^ T cells was determined using flow cytometric analysis, which was described in our previously protocol [15].

### Statistical Analysis

All data were analyzed using SPSS software version 19.0 (SPSS Inc., Chicago, IL, USA). Continuous variables were analyzed using a Mann–Whitney test or Kruskal–Wallis test. Paired-related data were analyzed using the Wilcoxon paired test. The correlation between two parameters was determined using Spearman’s bivariate correlation. Categorical variables were analyzed using a χ^2^-test or Fisher’s exact test. A *P* value of <0.05 was considered statistically significant.

## Results

Patient characteristics and laboratory data at the time of study entry (baseline) and at weeks 24 and 72 (time points for the beginning and end of SC) are summarized in Table 1. There was a significant decline in HBsAg levels from baseline to week 24 after commencing TDF (4.15 vs. 3.15, log10 IU/ml, *P* < 0.001), and this decline was observed in SC but not in non-SC patients. Moreover, there was a significant reduction in ALT levels from weeks 24 to 72 during TDF treatment (29.7 vs. 19.4 U/L, *P* = 0.03) as was observed in just SC patients. In addition, no other liver-related complications occurred during treatment, and no serious adverse events were identified. None of the patients experienced clearance of HBsAg during the course of therapy.

**Table 1:** Patient characteristics and laboratory values at baseline, 24W and 72W

**Characteristic**	**SC**			**Non-SC**		
	**Baseline**	**24W**	**72W**	**Baseline**	**24W**	**72W**
Age (years)	29.8 ± 8.9	-	-	34.4 ± 9.1	-	-
Gender (M/F)	7/2	-	-	9/2	-	-
ALT (U/L)	240.2 ± 67.8^a^	29.7 ± 11.0	19.4 ± 9.0*	156.7 ± 41.9^a^	48.2 ± 23.4	48.6 ± 35.9^▲^
TBiL (μmol/L)#	19.3 ± 8.8^a^	14.4 ± 4.9	12.7 ± 4.7	13.1 ± 3.7^a^	14.4 ± 3.2	11.6 ± 4.3
ALB (g/L)#	44.3 ± 1.8	47.3 ± 2.7	47.3 ± 0.7	45.3 ± 2.1	48.3 ± 3.3	47.4 ± 3.2
HBV DNA (log10 cps/ml)^§^	8.91 ± 0.56	4.07 ± 1.32	2.1 ± 0.04	8.46 ± 0.73	4.06 ± 1.30	2.53 ± 0.50
HBsAg (log10 IU/ml)	4.15 ± 0.53	3.15 ± 0.25**	2.93 ± 0.42	3.86 ± 0.91	3.43 ± 0.73^▲^	3.16 ± 0.41
HBV genotype	5B, 4C	-	-	7B, 4C	-	-

These values expressed as mean ± SD, unless otherwise indicated.

Normal values: ALT, ≤ 48 U/L; TBiL, ≤ 22 μmol/L; ALB, 32 ~ 50 g/L.

*P <0.05, comparing with week 24; **P <0.001, comparing with baseline; ^▲^*P* >0.05, comparing with baseline or week 24.

^§^*P* <0.001, among SC or non-SC groups; #*P* >0.05, among SC or non-SC groups.

^a^*P* <0.001, comparison between SC and non-SC groups at baseline.

ALT, alanine transaminase; ALB, albumin; DNA, deoxyribonucleic acid; HBV, hepatitis B virus; TBiL, Total bilirubin; −, useless; non-SC, non HBeAg seroconversion; SC, HBeAg seroconversion.

All subjects were of Chinese Han ethnicity.

### Changing HBsAg level in SC and non-SC patients

**Figure 1:**
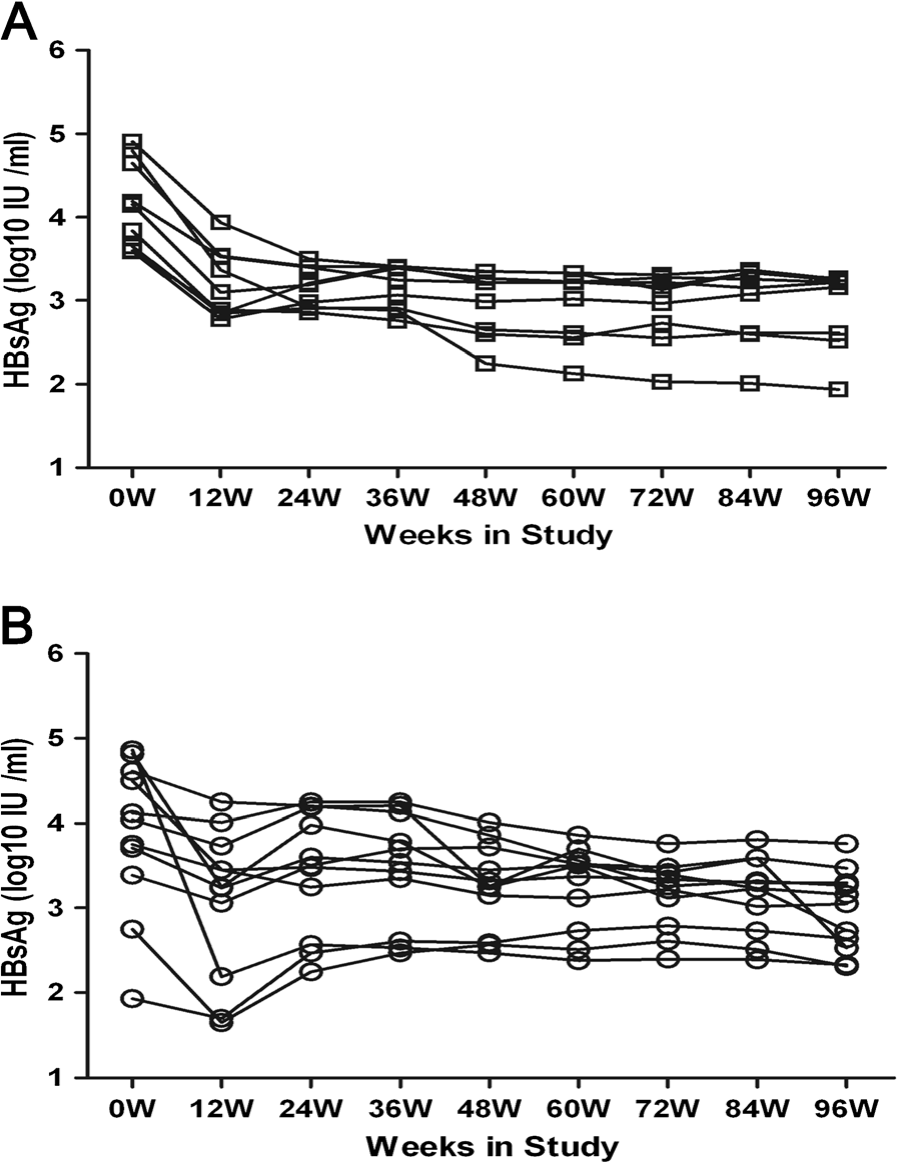
**Changing characteristic of HBsAg in SC or non-SC patients during treatment.** The changing profile of serum HBsAg (log10 IU/ml) level of each patient was classified to SC **(A)** or non-SC patients **(B)** during TDF treatment for 96 weeks.

We performed longitudinal analysis of HBsAg (log10 IU/ml) in peripheral blood in the SC and non-SC groups during 96 weeks of treatment with TDF. We found that the HBsAg content for each patient consistently declined over the course of 96 weeks of antiviral treatment period in the SC group (Figure 1A), which was different from the pattern of fluctuating decline in HBsAg levels in the non-SC group (Figure 1B). Furthermore, there were no significant differences between the SC and non-SC groups in the mean HBsAg levels at baseline or each treatment time point over the course of 96 weeks (Additional file 1: Figure S1). However, mean HBsAg was significantly different between the baseline and at week 12 in SC patients, but not in the non-SC group (Additional file 2: Figure S2).

### The relationship between serum HBsAg and HBeAg and ALT levels in SC and non-SC patients

**Figure 2:**
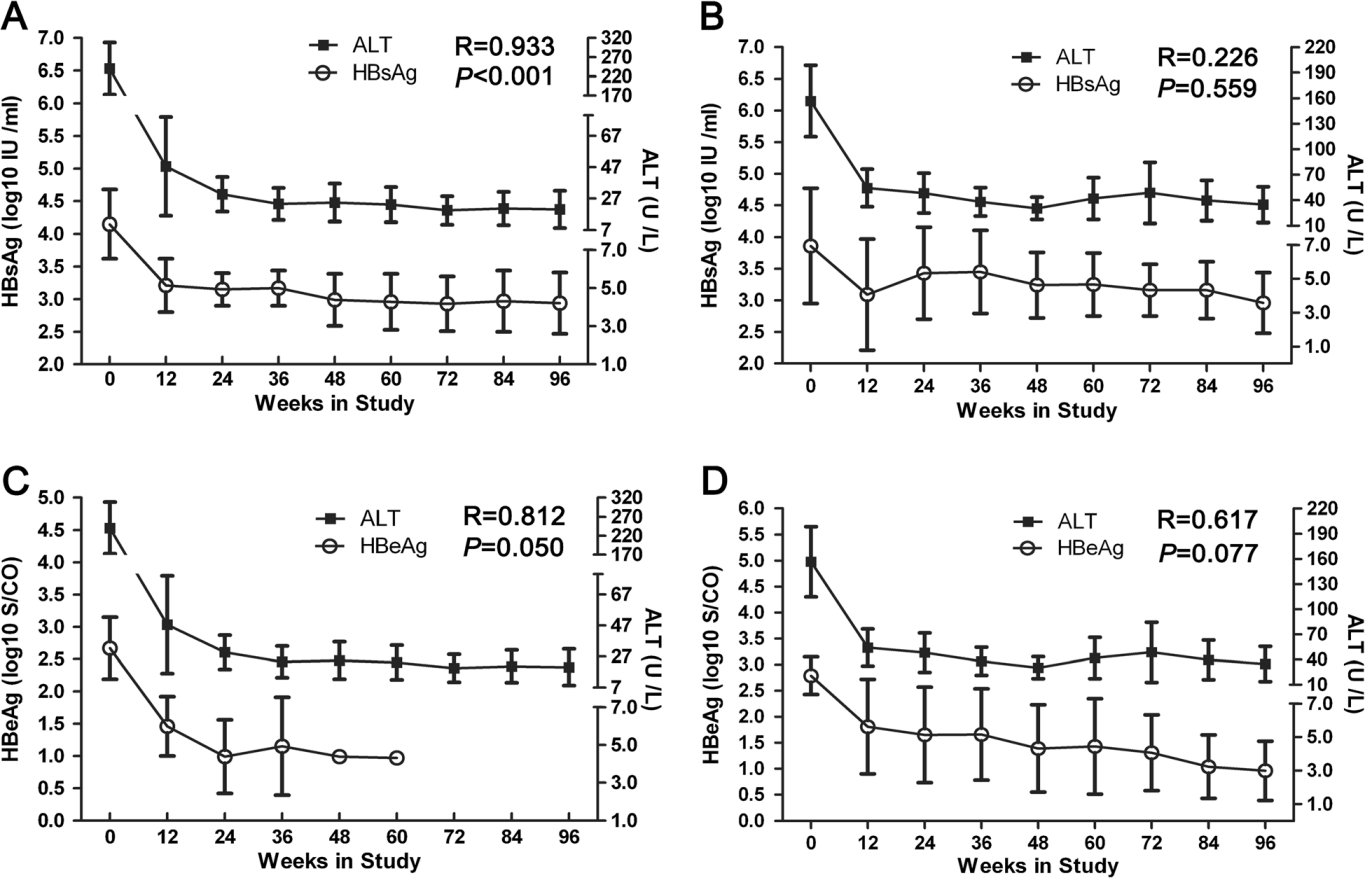
**Association between HBsAg or HBeAg and ALT levels in SC or non-SC patients during treatment.** The relationship between serum HBsAg (log10 IU/ml) and ALT (U/L) level is shown for SC **(A)** and non-SC patients **(B)**. The relationship between serum HBeAg (log10 S/CO) and ALT (U/L) level is shown for SC **(C)** and non-SC patients **(D)**. Correlations were analyzed using Spearman correlation analysis.

In SC patients, there was significant reduction in the serum ALT level from baseline to week 12 (mean, 240.22 to 47.44 U/L). In addition, there was a continued progressive reduction in HBsAg levels during therapy compared to the baseline (Figure 2A). However, a fluctuating decline in HBsAg and ALT levels was observed in non-SC patients (Figure 2B). Moreover, SC patients showed a significant correlation between changes in HBsAg and ALT levels (R = 0.933, *P* < 0.001). No significant correlation was observed in the non-SC group, however (R = 0.226, *P* = 0.559).

In a manner similar to the association between serum HBsAg and ALT levels in the non-SC group, the HBeAg level declined in SC-patients during the course of treatment along with ALT, and there was no significant relationship between them (R = 0.812, *P* = 0.050; Figure 2C). A similar relationship was found in the non-SC group as well (R = 0.617, *P* = 0.077; Figure 2D).

### The relationship between serum HBsAg and HBeAg and circulating HBV DNA levels in SC and non-SC patients

**Figure 3:**
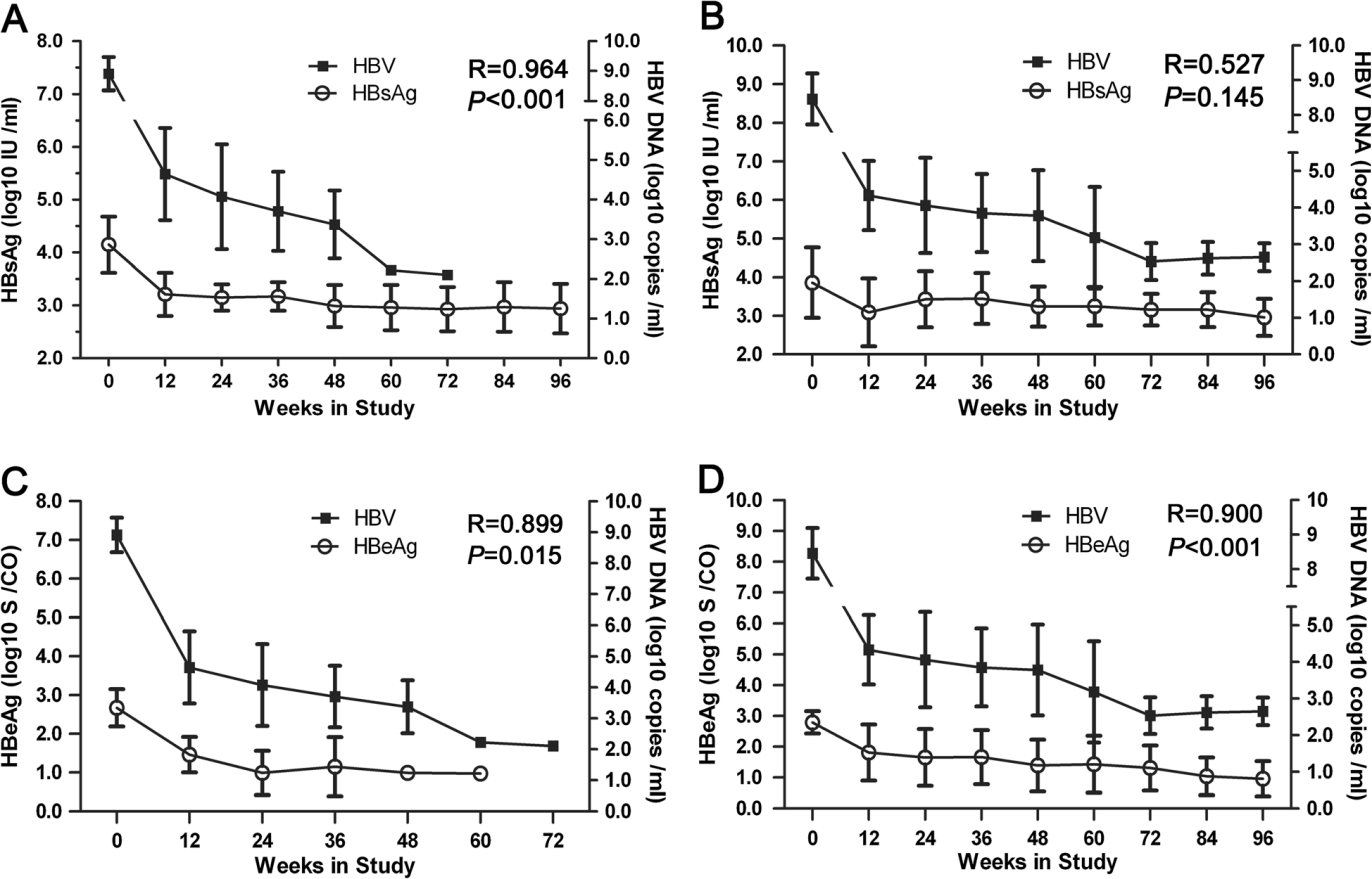
**Association between HBsAg or HBeAg and HBV DNA level in SC or non-SC patients during treatment.** The relationship between serum HBsAg (log10 IU/ml) and HBV DNA level (log10 copies/ml) is shown for SC **(A)** and non-SC patients **(B)**. The relationship between serum HBeAg (log10 S/CO) and HBV DNA level (log10 copies/ml) is shown for SC **(C)** and non-SC patients **(D)**. Correlations were analyzed using Spearman correlation analysis.

As was the case for the association between serum HBsAg and ALT levels in the SC group, the HBsAg level declined along with HBV DNA in the SC group over the course of treatment, and there was a significant relationship between them (R = 0.964, *P* < 0.001; Figure 3A). However, a significant positive relationship was not observed in non-SC patients (R = 0.527, *P* = 0.145; Figure 3B). In addition, in the SC group, no HBeAg could be detected after week 60, and no HBV DNA could be detected after week 72. Furthermore, there was a significant relationship between these two parameters (R = 0.899, *P =* 0.015; Figure 3C). In non-SC patients undergoing TDF treatment, the fluctuating reduction in HBV DNA loading was concurrent with a stable decline in HBeAg, and there was significantly relationship between these two parameters as well (R = 0.900, *P <* 0.001; Figure 3D).

A significant correlation was observed between HBsAg (log10 IU/ml) and HBV DNA (log10 copies/ml) in all HBeAg-positive patients (SC and non-SC patients) at baseline, but no significant correlation was observed at week 24 (Additional file 3: Figure S3). Simultaneously, a significant correlation between HBsAg and HBV DNA loading was a prominent discrepancy between SC and non-SC patients at baseline (Additional file 4: Figure S4).

### The relationship between serum HBsAg and HBeAg levels and circulating Treg frequencies in SC and non-SC patients

**Figure 4:**
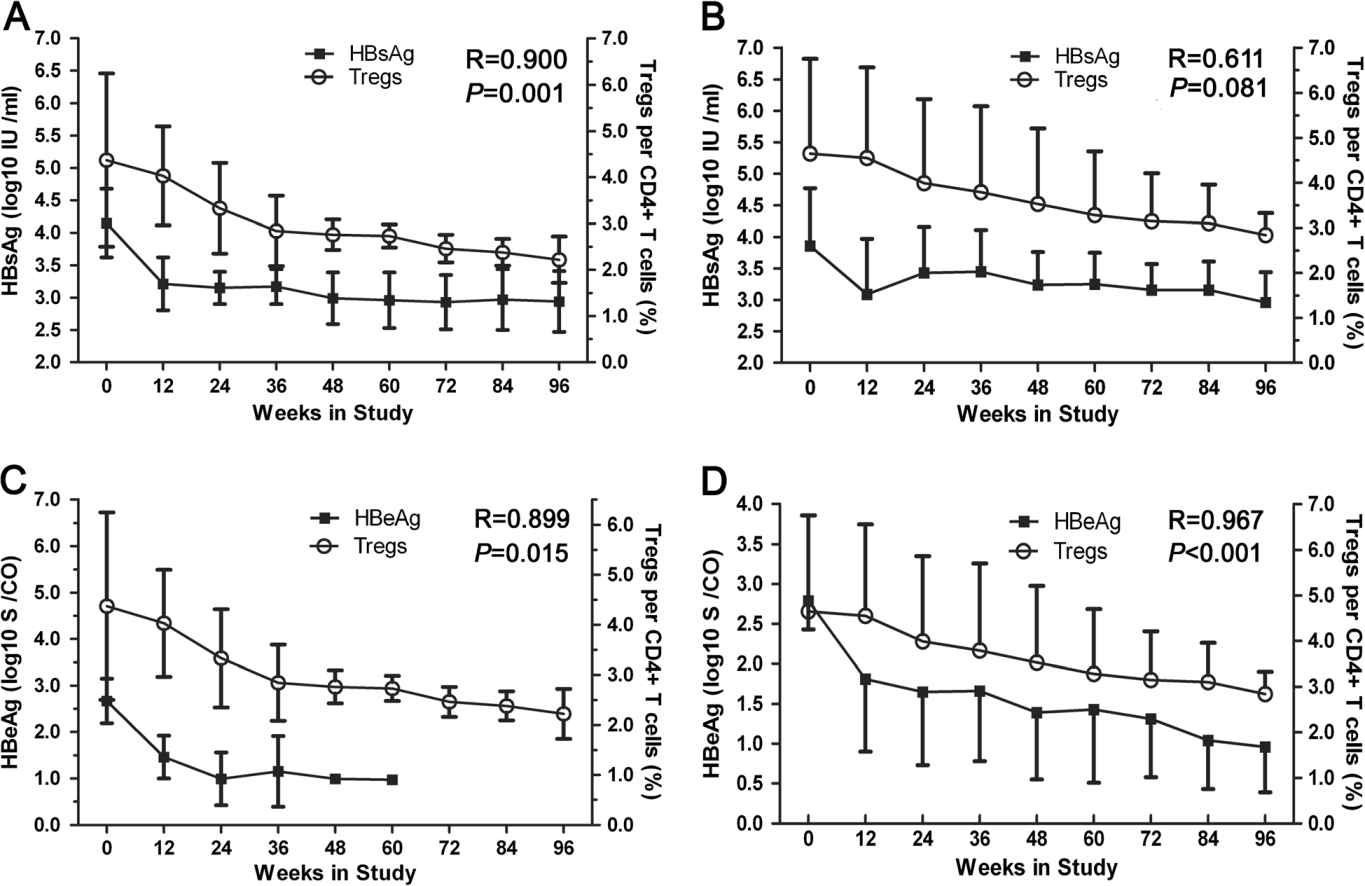
**Association between HBsAg or HBeAg and Treg frequencies in SC or non-SC patients during treatment.** The relationship between serum HBsAg (log10 IU/ml) level and circulation CD4^+^CD25^+^ Treg frequency is shown for SC **(A)** and non-SC patients **(B)**. The relationship between serum HBeAg (log10 S/CO) level and circulation CD4^+^CD25^+^ Treg frequency is shown for SC **(C)** and non-SC patients **(D)**. Correlations were analyzed using Spearman correlation analysis.

As was observed with the changing of serum HBsAg levels associated with HBV DNA loading, circulating Treg frequencies showed a very steady decline along with the HBsAg level decline in the SC group during TDF treatment, and there was a significant relationship between these two parameters (R = 0.900, *P* = 0.001; Figure 4A). However, in the non-SC group, the Treg frequency declined stably along with a fluctuant reduction in the HBsAg level during treatment, and the turning point for the declining HBsAg level was observed at week 12. In addition, there was no significance between HBsAg and HBV DNA loading (R = 0.611, *P* = 0.081; Figure 4B).

As was the case for changes in the serum HBsAg level, the Treg frequencies declined along with a decline in the HBeAg level during treatment of patients in the SC group, and there was a significantly relationship between the two parameters (R = 0.899, *P* = 0.015; Figure 4C). Similarly, there was a significant difference between the circulating Treg frequency and the HBeAg level in the non-SC group as well (R = 0.967, *P* < 0.001; Figure 4D).

### The relationship between serum HBsAg and HBeAg levels in SC and non-SC patients

**Figure 5:**
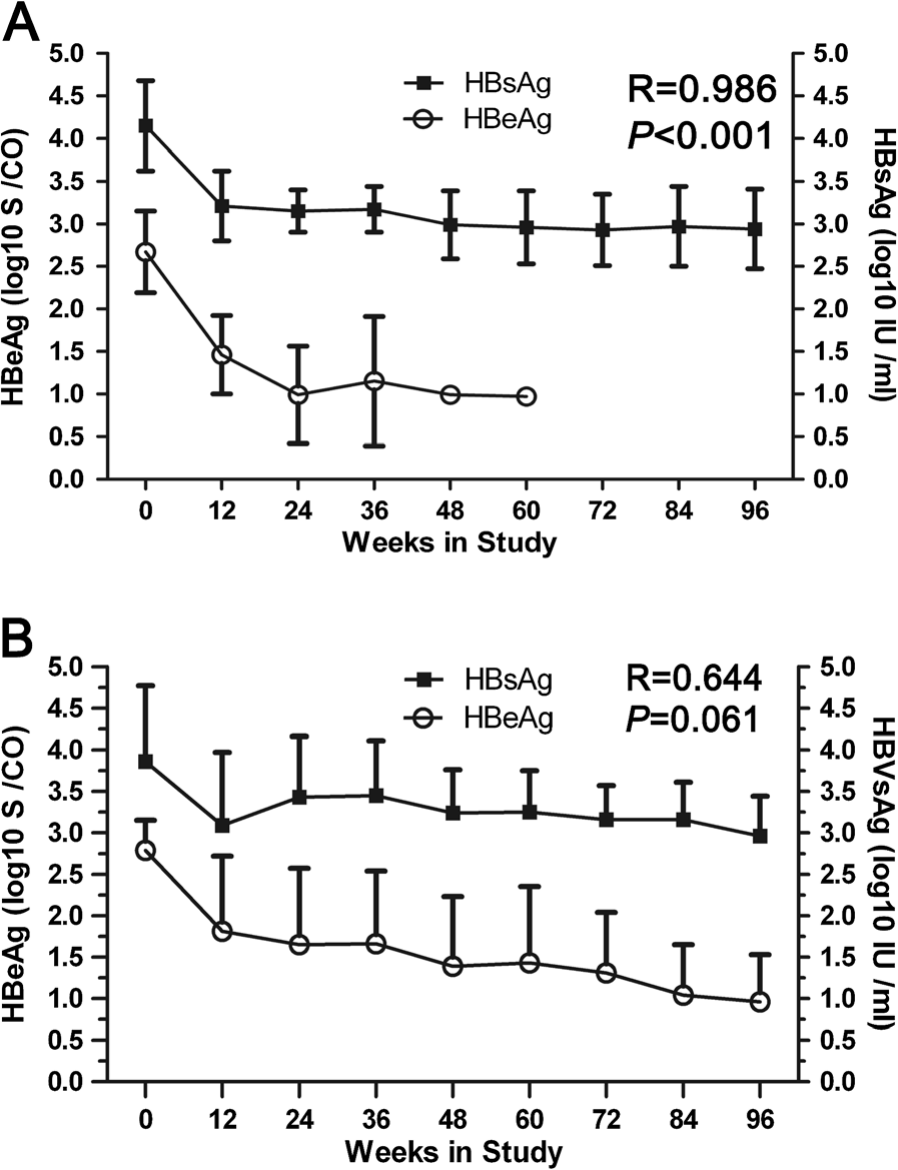
**Association between HBsAg and HBeAg in SC or non-SC patients during treatment.** The relationship between serum HBsAg (log10 IU/ml) and HBeAg (log10 S/CO) level is shown for SC **(A)** and non-SC patients **(B)**. Correlations were analyzed using Spearman correlation analysis.

We observed a significant positive correlation between HBsAg and ALT, HBV DNA loading, and Treg frequencies (*P* < 0.001, *P* < 0.001, or *P* = 0.001, respectively) in SC patients during the TDF therapy period. We also observed a significant positive relationship between HBsAg and HBeAg (R = 0.986, *P* < 0.001; Figure 5A). However, a significant relationship was not present in non-SC patients (R = 0.644, *P* = 0.061; Figure 5B). Furthermore, there was no significant relationship between HBsAg and HBeAg in any patients prior to antiviral treatment (data no shown).

## Discussion

Measurement of serum HBV DNA loading remains as important tool for monitoring antivral treatment outcomes for CHB patients who do not always experience SC, although they have undergone with potently suppress HBV replication, followed by treatment with regular antiviral treatment [17-19]. However, HBsAg clearance from the serum of CHB patients usually indicates clinical cure and is associated with improved survival. In recent years, increasing attention has been paid to the quantitation of HBsAg and the use of this knowledge in the antiviral treatment of CHB patients [12,20,21]. The present study indicated that quantitated HBsAg serves as a useful marker for response to antiviral therapy in SC and non-SC patients during TDF treatment.

At present, HBsAg has been developed as an alternative marker for the monitoring of antiviral treatment outcomes for CHB patients [20,22]. In fact, HBsAg production does not correlate closely with viral replication because it depends on transcriptional activities involving other enzymatic factors [23]. The production of HBsAg is in excess to whole virions (HBV Dane particles) because serum HBsAg includes two other elements (small spherical and tubular particles). Therefore, the correlation between HBV DNA and HBsAg levels may be imbalanced, indicating that antiviral therapy does not directly influence the serum HBsAg level under some conditions [22].

In the current study, our results are consistent with the hypothesis that there was no significant correlation between serum HBsAg and HBV DNA levels in all HBeAg positive patients over the course of TDF treatment, however, there was a different significant correlation during antiviral treatment when the relationship between HBsAg and HBV DNA levels were analyzed separately in SC and non-SC patients, respectively. The correlation for SC patients was significant, while the correlation for non-SC patients was not. These results suggested that serum HBsAg levels could reflect the HBV DNA level in undergoing SC CHB patients during TDF treatment, which is consistent with the hypothesis that quantitative serum HBsAg has a high predictive value for treatment response towards IFNα-1b or entecavir [21,24]. Furthermore, the relationships between HBeAg and ALT (HBV DNA, or Tregs) in SC or non-SC patients during treatment were similar statistical relationship (both are significant relationship or not), which suggests that quantitative HBsAg is a more useful indicator than HBeAg for distinguishing between SC and non-SC patients during TDF treatment. This is in contrast to Singh, A. et al’s findings that indicated that the HBsAg decline did not correlate with HBeAg seroconversion in HBeAg-positive patients, and reduction in HBV DNA levels at week 4 and 12 correlated with seroconversion [25]. This difference may be related to the opinion that the variable pattern and clinical outcome of HBV infection were mainly determined by virological itself factors, host immunological factors and genetic factors of host [26].

Additionally, the positive relationship between HBsAg and ALT levels was significant in SC but not in non-SC patients, further indicating the importance of analyzing HBsAg levels during TDF treatment.

SC is an indicator of positive outcomes for HBeAg-positive CHB patients subjected to antiviral treatment, and the SC process is affected by many factors. Our results showed that SC patients had a significantly higher serum ALT level at baseline than did non-SC patients (Table 1), which was partially consistent with Tseng’s report that a higher serum ALT level correlated with earlier SC in lamivudine-treated chronic hepatitis B patients [27]. In addition, we also discovered that the serum TBiL level at baseline was higher in SC than in non-SC patients (Table 1).

It is generally believed that serum ALT levels reflect the host immune response to chronic HBV infection, and higher ALT levels indirectly indicate that SC patients are more readily restored to effective immune function than non-SC patients. An intensive immune response is quickly restored after HBV replication has been inhibited during antiviral treatment, reducing damage to liver cells and restoring serum ALT levels to normal [28-30]. In our study, the ALT level rapidly declined to a normal level (<46 U/L) at week 12 in SC patients during treatment, but did not in non-SC patients, which most likely correlates with the significant decline in HBsAg that was observed in CHB patients with preexisting immune activity, as was reflected by high baseline ALT levels [11,31,32]. Although, the HBsAg level in non-SC patients was depressed at the beginning of treatment, these levels rebounded over the course of treatment.

Tregs play a key role in the maintenance of immune tolerance and regulation of the immune response [33]. Tregs numbers typically increase in CHB patients and are restored to normal levels in recovered CHB patients subjected to antiviral treatment [34]. The results of this study showed a significant positive correlation between HBsAg and Treg levels in SC patients during treatment, which further confirms that a reduction in Treg numbers is related to the process of SC, and patients who did not achieve SC may be associated without being restored to normal Treg numbers.

The immune mechanisms involved in SC are still not fully understood. A high HBeAg level is believed to induce T-cell tolerance or hyporesponsiveness [35]. During antiviral treatment, specific enhancement of the cellular immune response is indirectly caused by the suppression of HBV replication and a reduction in the HBeAg antigen level, rather than as a direct effect of any particular drug. In addition, it has been reported that even high concentrations of entecavir had no significant effect on the survival and function of immune cells in vitro [36], and antiviral therapy can promote a reduction in higher PD-1 levels caused by a high HBeAg level and thus elevate the level of HBV-specific CTL [37]. In this study, serum HBsAg levels began to decline in response to anti-viral treatment, and hence circulating Tregs were restored to normal levels. This process reduced the inhibitory affect on antigen-specific T cells and promoted HBeAg SC process. These results confirmed previous observations that immune modulation is of vital importance for the complete eradication of HBV and HBeAg seroconversion [38,39]. In addition, the significant positive relation between HBsAg and HBeAg levels in SC but not in non-SC patients during antiviral treatment further implies that quantitating HBsAg is more importance for the determination of seroconversion status during TDF treatment, and HBsAg may be an immunomodulatory molecule over the course of CHB [40].

Limitations for this study include a short follow-up period and the fact that none of SC patients lost HBsAg. Therefore, the predictive value of quantitating HBsAg levels for antiviral treatment-induced HBsAg seroconversion could not be evaluated. Previous studies have shown that HBsAg suppression in response to interferon-based regimens may be predictive of subsequent HBsAg clearance and seroconversion [10,41]. However, only a minority of interferon-treated patients lose their HBsAg. One possible explanation for the difference in HBsAg kinetics is that PEG-IFN has a remarkable immune regulatory effect and TDF has significant effects on viral replication, but a minimal effect on the immune response [12]. Therefore, it may take longer for the host to re-initiate the immune response and demonstrate a decline in the HBsAg level. These results indirectly verified that long-term antiviral therapy is very necessity for patients with CHB, and suggest that the HBsAg level is closely association with the immune status of CHB patients [40].

Another limitation of our study is that our patient cohort was small, cccDNA levels, and baseline drug-resistance mutational analysis were not measured, simultaneously. Indeed, cccDNA may become the dominant form of HBV DNA during viral suppression with entecavir and may be responsible for ongoing production of HBsAg [42]. Reijnders, J. et al.’s multivariate analysis showed that a decline in HBsAg was only related to the treatment regimen (*P* <0.001) and not to any other baseline variable [10].

## Conclusions

In summary, without considering the factor of 58 of the 78 patients dropped out in this study, close to half of patients treated with TDF for 96 weeks achieved SC between weeks 24 to 72. A significant, early decline in HBsAg at 12 weeks was associated with higher rates of complete HBV DNA suppression and SC during the TDF treatment period. Therefore, the quantitation of HBsAg is a more useful indicator than HBeAg for distinguishing SC from non-SC patients during TDF treatment.

## Abbreviations

ALT: alanine transferase; ALB: albumin; cccDNA: covalently closed circular DNA; CHB: chronic hepatitis B; DNA: deoxyribonucleic acid; HBsAg: hepatitis B surface antigen; HBeAg: hepatitis B e antigen; HBV: hepatitis B virus; non-SC: non HBeAg seroconversion; SC: HBeAg seroconversion; TBiL: Total bilirubin; TDF: tenofovir disoproxil fumarate; Treg: CD4^+^CD25^+^ regulatory T cell.

## Competing interests

The authors declare that they have no competing interests.

## Authors’ contributions

YJZ contributed to the study design, data collection, most experiments, and the writing the initial draft and revising the manuscripts. CJJ and YP collected the preliminary data, and helped to perform some experiments. JLF participated in the study design and interpretation of the data. WW and SGP assisted in experimental design and help to data collection. LLJ contributed to the study coordination, technical issues and revision of the manuscript. All authors read and approved the final manuscript.

## Supplementary Material

Additional file 1: Figure S1 Changing HBsAg level in SC and non-SC patients during treatment. Serum HBsAg (log10 IU/ml) level in SC and non-SC patients during TDF treatment for 96 weeks was shown, respectively. Click here for file

Additional file 2: Figure S2 HBsAg varying at baseline and week 12 in SC and non-SC patients during treatment. The serum HBsAg level (log10 IU/ml) was compared between at baseline and week 12 in SC and non-SC patients during treatment, respectively. Click here for file

Additional file 3: Figure S3 The association between HBsAg and HBV DNA level in all HBeAg positive patients. The relationship between serum HBsAg (log10 IU/ml) and HBV DNA (log10 copies/ml) level in all HBeAg positive patients is shown at baseline (A) and week 24 during treatment (B). Click here for file

Additional file 4: Figure S4 The association between HBsAg or HBV DNA levels in SC or non-SC patients at baseline. The relationship between serum HBsAg (log10 IU/ml) and HBV DNA (log10 copies/ml) level is shown for SC (A) and non-SC patients (B) at baseline. Click here for file
