# Supplementary figures and images for: HBsAg as an important predictor of HBeAg seroconversion following antiviral treatment for HBeAg-positive chronic hepatitis B patients
Source: J Transl Med. 2014 Jun 25;12:183. doi: 10.1186/1479-5876-12-183 (PMC4230803; doi:10.1186/1479-5876-12-183)

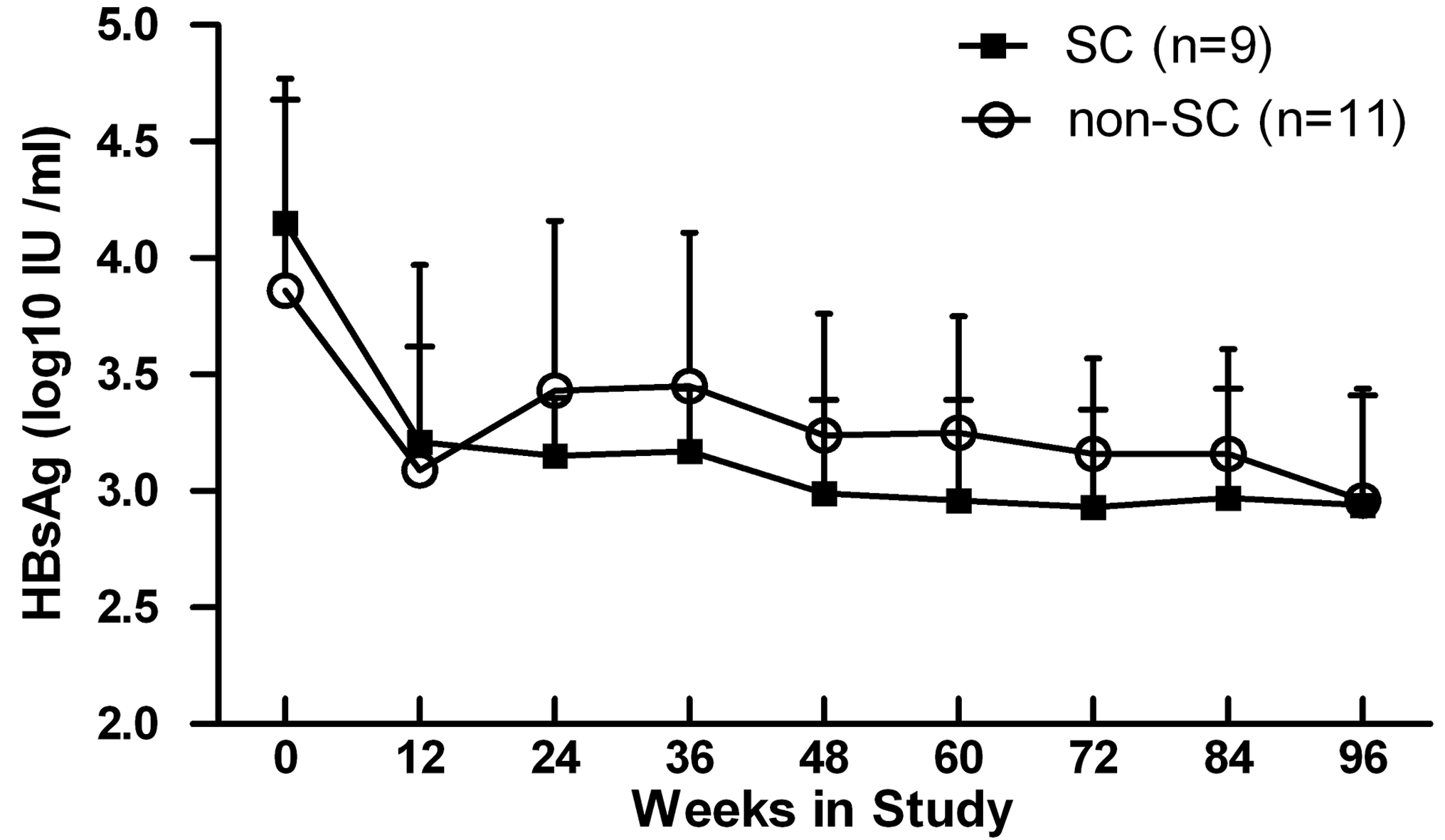

Supplement: Additional file 1: Figure S1 — Changing HBsAg level in SC and non-SC patients during treatment. Serum HBsAg (log10 IU/ml) level in SC and non-SC patients during TDF treatment for 96 weeks was shown, respectively. [file 1479-5876-12-183-S1.tiff]

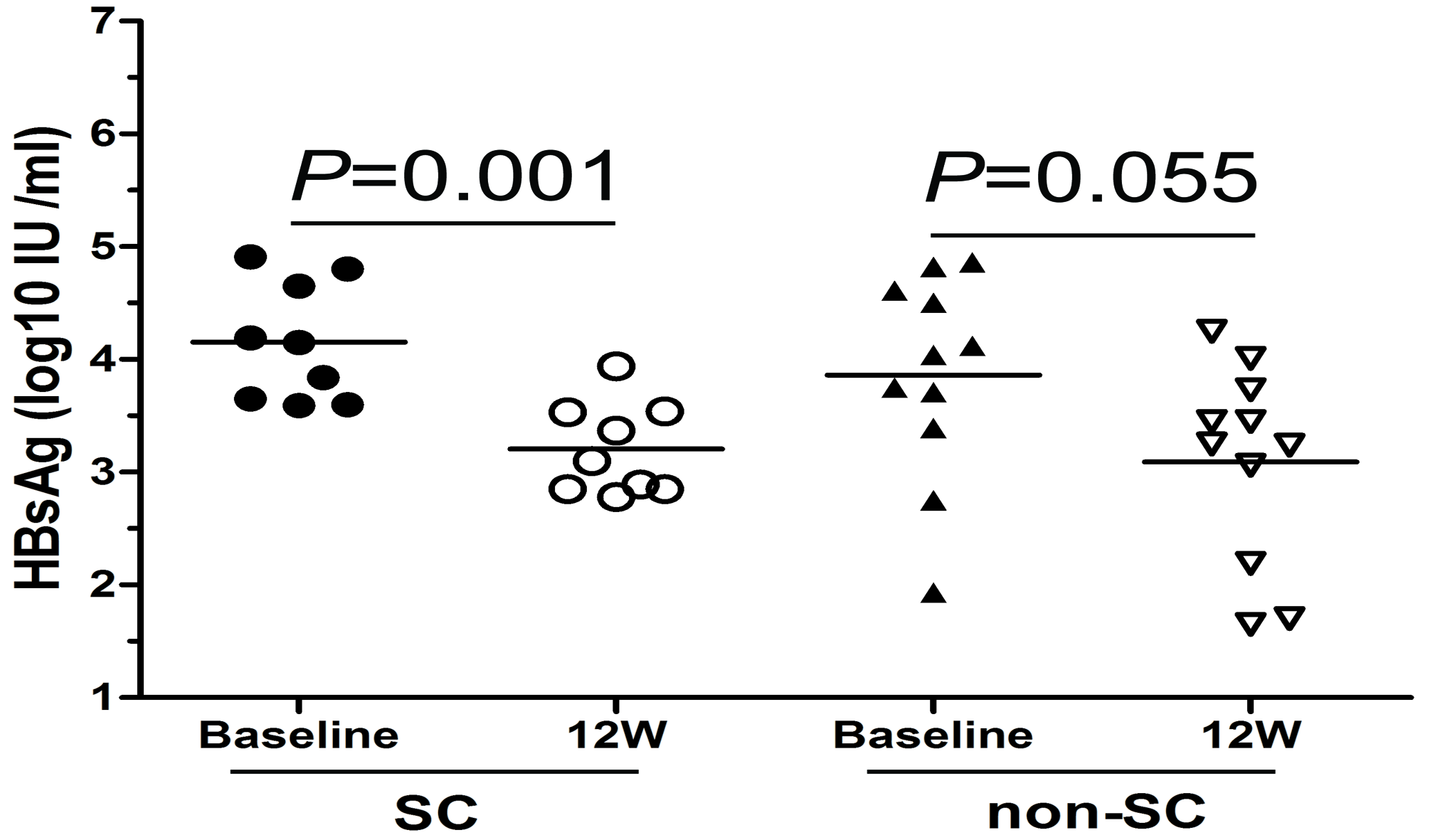

Supplement: Additional file 2: Figure S2 — HBsAg varying at baseline and week 12 in SC and non-SC patients during treatment. The serum HBsAg level (log10 IU/ml) was compared between at baseline and week 12 in SC and non-SC patients during treatment, respectively. [file 1479-5876-12-183-S2.tiff]

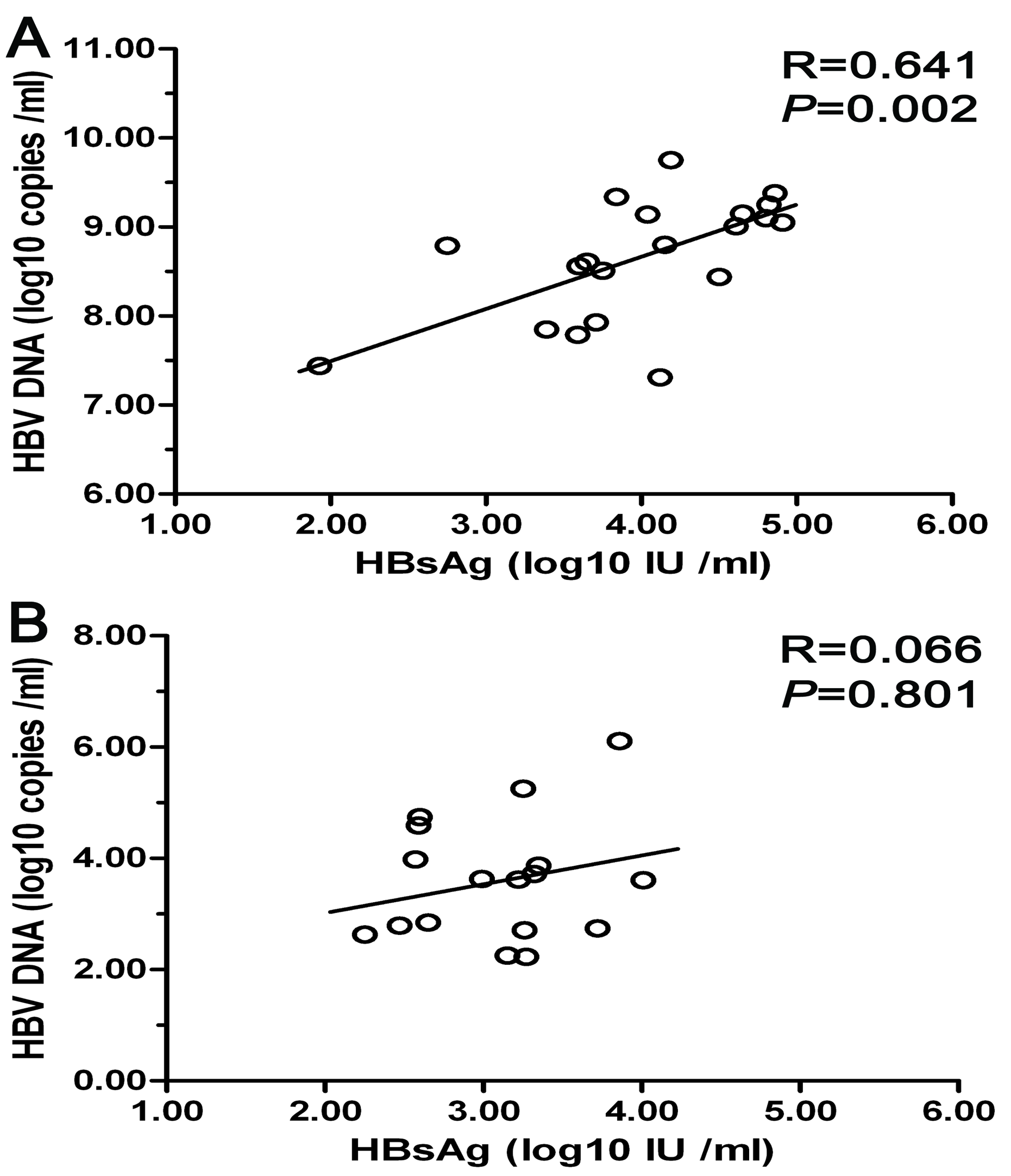

Supplement: Additional file 3: Figure S3 — The association between HBsAg and HBV DNA level in all HBeAg positive patients. The relationship between serum HBsAg (log10 IU/ml) and HBV DNA (log10 copies/ml) level in all HBeAg positive patients is shown at baseline (A) and week 24 during treatment (B). [file 1479-5876-12-183-S3.tiff]

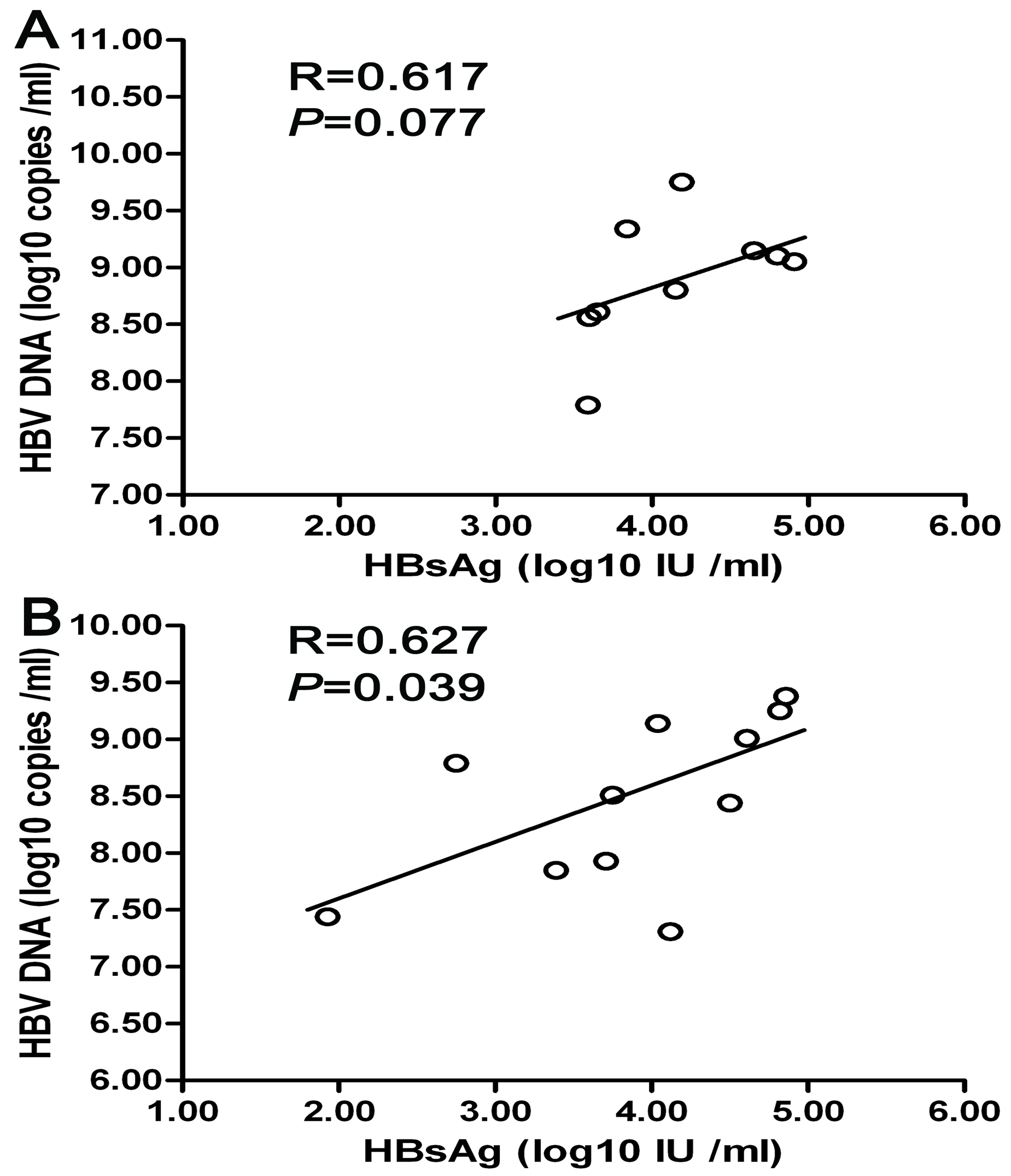

Supplement: Additional file 4: Figure S4 — The association between HBsAg or HBV DNA levels in SC or non-SC patients at baseline. The relationship between serum HBsAg (log10 IU/ml) and HBV DNA (log10 copies/ml) level is shown for SC (A) and non-SC patients (B) at baseline. [file 1479-5876-12-183-S4.tiff]
